# Diseases and Mortality in Confiscated Birds and Reptiles Housed in a Wildlife Rescue Center Under the CITES Directive

**DOI:** 10.3390/vetsci13030306

**Published:** 2026-03-23

**Authors:** Aurora Martín, Adrián Rabanal Soto, Víctor Hidalgo-Martínez, Adriana Rodríguez Luis, María del Carmen Aranda Vázquez, Paloma Jimena de Andrés Gamazo, María de los Ángeles Jiménez Martínez

**Affiliations:** 1 Department of Medicine and Surgery, Veterinary Faculty, Complutense University of Madrid, 28040 Madrid, Spain; aumart02@ucm.es (A.M.); adraba01@ucm.es (A.R.S.); victorhi@ucm.es (V.H.-M.); marara07@ucm.es (M.d.C.A.V.); pjandres@ucm.es (P.J.d.A.G.); 2 FIEB Foundation (Foundation for Research in Ethology and Biodiversity), 45112 Toledo, Spain; adriana.rodriguez@fiebfoundation.org; 3 Tragsatec, 28006 Madrid, Spain

**Keywords:** infectious disease, hemosporozoa, septicemia, renal gout, health monitoring, FIEB Foundation

## Abstract

The number of confiscated CITES-listed animals has increased dramatically worldwide, creating significant health, logistical, and resource challenges for responsible authorities. Rescue centers represent a scientific and humanitarian response to this challenge, providing solutions through rehabilitation, research, and environmental education. This postmortem survey provides information on disease and mortality during a four-year period, in confiscated CITES-listed birds and reptiles housed in an authorized rescue center. A total of 29 animals (17 birds and 12 reptiles) were examined by necropsy and histopathology. Infectious disease accounted for the mortality of 58.8% of birds and 49.8% of reptiles, with overrepresentation of bacterial disease in both groups. Lesions consisted mainly of granulomas in multiple organs. Suspected viral disease occurred in 23.3% of birds, and protozoal infections were found in 17.3% of birds. Systemic disease caused by an unknown haemosporozoan was the cause of death in a *Lonchura oryzivora*. An unknown infectious agent was associated with renal disease in a *Ctenosaura* sp. Gout secondary to dehydration was overrepresented in reptiles (33.3%). This study highlights the complexity of disease processes affecting confiscated birds and reptiles in CITES rescue settings and provides invaluable information for other rescue centers that may impact the success of conservation strategies.

## Simple Summary

The global rise in confiscated animals protected under CITES has created significant health and resource challenges for wildlife authorities. Rescue centers play a key role by caring for these animals, supporting rehabilitation, and contributing to research and education. This study reviewed the causes of illness and death over four years in confiscated birds and reptiles in an authorized rescue center and determined that the leading cause of death was infectious disease in over half of the animals. Bacterial infections were most common, often affecting multiple organs. Some birds also showed signs of viral or parasitic infections, including one fatal case caused by a blood parasite. In reptiles, dehydration-related gout was a frequent finding, and one case involved kidney disease linked to an unidentified infectious agent. The results highlight the complex health problems faced by confiscated wildlife and emphasize the need for improved management and more individualized medical care in rescue centers.

## 1. Introduction

Illegal wildlife trade has profound environmental, social, and economic repercussions, causing severe losses in biodiversity that threaten endangered species and undermine the stability of entire ecosystems [1,2]. The Convention on International Trade in Endangered Species of Wild Fauna and Flora (CITES) is the international framework responsible for regulating trade in endangered species. Among the fundamental principles of the Convention are two lists of species (Appendix I and Appendix II) that require strict regulations for manipulation and trade to safeguard their survival. Appendix I includes species threatened with extinction that are or may be affected by trade, whereas Appendix II includes specimens not necessarily threatened with extinction but for which trade must be strictly regulated to prevent endangerment [1,2]. The Convention establishes specific regulations that are incorporated into domestic legislation, forming laws and programs designed to prevent abuse and illegal trafficking. These regulations encompass the trade of live specimens and include enforcement measures that penalize illegal trade and possession, as well as confiscation or repatriation to the country of origin. Confiscated specimens are entrusted to the Management Authority, which will safeguard the specimen either through return to the country of origin or placement in a designated rescue center. A rescue center is defined as an institution appointed by the Management Authority to safeguard the welfare of confiscated living specimens [2].

Thousands of species are traded as pets, many of which have been subject to inadequate regulation, contributing to the extinction of several reptile species and severe declines in bird populations [1,2,3]. In parallel, the number of confiscated live animal specimens has increased dramatically in recent years worldwide, creating significant health, logistical, and resource challenges for responsible authorities. This situation has demanded the implementation of stricter quarantine protocols, specialized treatments, and enhanced sanitary management to prevent the spread of disease [1].

Rescue centers represent a scientific and humanitarian response to this challenge, providing solutions through rehabilitation, research, and environmental education. However, many centers struggle to provide appropriate housing, management, and long-term care for confiscated animals, which often require prolonged stays and adaptable management strategies [1,4]. In Spain, the Foundation for Research in Ethology and Biodiversity (FIEB) is a wildlife biodiversity research and rehabilitation center, designated by the Spanish authorities as a CITES rescue center.

Taxa-specific information on pathologies or medical management is often scarce or too general in scope, particularly when it comes to birds and reptiles. This presents a substantial challenge for wildlife and CITES-approved rescue centers that take in and maintain a broad number of taxa, often for unknown and prolonged periods of time. Birds and reptiles raise challenges in captivity, given their vast anatomical variations and specific biological and physiological characteristics [5]. Additionally, many species are prone to being extremely sensitive to stressful environmental situations and changes, which warrants minimal and precise manipulation in rescue centers [4,6,7]. Because of this, postmortem studies are an invaluable source of information [8].

Retrospective morbidity and mortality studies in zoological and captive wildlife populations provide useful information on diseases and welfare of animals that is otherwise elusive. These studies prove to be essential tools for discerning common and uncommon diseases in taxa that are otherwise difficult to encounter and manage [8].

The purpose of this study was to add to the pool of existing data on common and uncommon diseases and taxa-specific entities in confiscated birds and reptiles, by surveying causes of mortality and diseases during a four-year period at a FIEB research center. The information provided will help enlighten medical advancements, management, and disease control strategies, making them applicable to other rescue centers offering sanctuary to confiscated animals and impacting the success of the rehabilitation and conservation strategies.

## 2. Materials and Methods

Between January 2021 and December 2024, 29 confiscated animals, comprising 17 birds and 12 reptiles, were submitted to necropsy at FIEB Wildlife Rescue Center. Details on the number of species are listed in Table 1.

Animals confiscated and submitted to FIEB are housed in isolation under quarantine for a period of 21 days to one month. After this period, they are either introduced to enclosures with conspecific individuals or transferred to new enclosures following adaptation protocols.

Aviaries are routinely cleaned with a pressure washer and disinfected twice a week. Crates and cages are emptied, disinfected, and placed under quarantine for three weeks after any suspicious or infectious cause of death, or until pathology results confirm clearance. Reptile enclosures are emptied and cleaned monthly, and the substrate is carefully inspected to determine whether it can be safely reused. Fecal examinations are conducted prior to release from quarantine and routinely once per semester. Treatment is initiated only upon positive diagnosis of parasites or other infections.

The FIEB research center received a total of 339 confiscated live animal specimens (25 mammals, 262 birds, and 52 reptiles) between 2021 and 2024, of which 105 were repatriated (sent to the country of origin) or reallocated to other centers or sanctuaries. All animals included in this study had been held at FIEB for a minimum of 2 months and a maximum of 11 years from the date of confiscation. Confiscation was produced by the competent authorities of the Spanish Government (Civil Guard and the Ministry for Ecological Transition and Demographic challenge) in compliance with the provisions of the CITES Convention.

Necropsies were performed by the center’s veterinarians following standard protocols provided by the European Association of Zoos and Aquaria to its members [9]. Tissue samples of lungs, liver, heart, kidney, spleen, intestines, pancreas, gastrointestinal tract (including proventriculus, ventriculus and crop in birds), gonads, and skin were harvested and fixed in 10% neutral buffered formalin.

Fixed samples were submitted to the Zoo and Wildlife Pathology Service of the Complutense University Teaching Hospital (HCVC), trimmed, embedded in paraffin, sectioned at 4 microns, and stained with hematoxylin and eosin (H&E) for routine histopathological examination under a light microscope. Special stains including Giemsa, Congo Red, Periodic Acid-Schiff (PAS), Masson’s trichrome, Ziehl-Neelsen (ZN) for acid-fast organisms were obtained when pertinent.

## 3. Results

Table 2 and Table 3 list the histopathological lesions within organ systems that were observed in birds and reptiles.

### 3.1. Systemic Lesions

**Figure 1:**
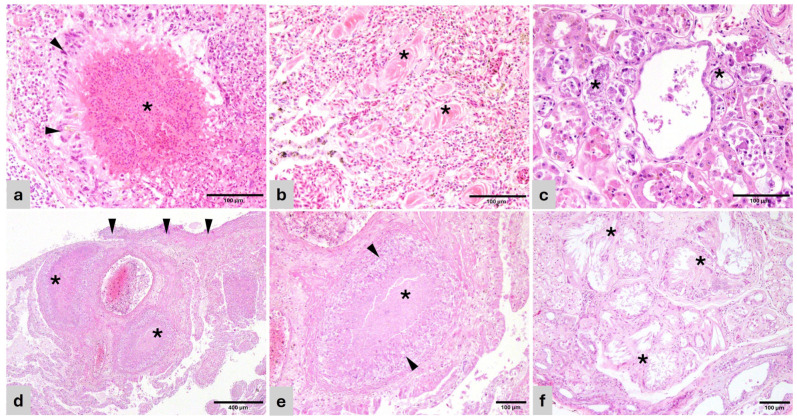
Histopathology of representative lesions (H&E). (**a**) Cacatua alba. Lung—Heterophilic granuloma with necrotic center and bacteria (*) surrounded by palisading epithelioid macrophages and giant multinucleated cells (arrowhead). (**b**) Lorius chlorocercus. Lung—Air capillaries disrupted by protein deposits (*) surrounded by collagenous matrix and fibroblasts (pulmonary proteinosis). (**c**) Amazona aestiva. Kidney—Renal tubules with effaced epithelium, replaced by pale basophilic and amphophilic, fragmented urate tophi (*). (**d**) *Uromastyx* sp. Lung—Foveal septae are disrupted by coalescing granulomas (*) and sheets of macrophages and heterophils. The pleura is expanded by similar inflammatory cells and bacterial colonies (arrowheads). (**e**) *Uromastyx* sp. Lung—Detail of a granuloma in the foveal interstitium with central necrosis (*) surrounded by palisading epithelioid macrophages and giant multinucleated cells (arrowheads). (**f**) Varanus pilbarensis. Kidney—Obliterated renal tubules are replaced by radiating crystals of urate tophi (*) surrounded by macrophages, giant multinucleated cells, and mature connective tissue.

In birds, pulmonary edema generally associated with congestion was the most frequent respiratory lesion (47%), followed by granulomatous pneumonia (23.5%). Edema and congestion were acute and diffuse while pulmonary granulomas were predominantly multifocal, consisting of well defined, nodular aggregates of macrophages, usually with a central area of necrotic debris and heterophils, surrounded by macrophages, palisading epithelial macrophages, and giant multinucleated cells (Figure 1a). All cases were negative for acid-fast bacteria, discarding mycobacteria. Pulmonary proteinosis and fibrosis were observed in the lung of one bird (5.8%) consisting of deposits of eosinophilic crystalline material predominantly within air capillaries and in lesser measure within adjacent bronchioles. The adjacent interstitium and the air capillary septae were replaced with fibrosis (Figure 1b).

Heterophilic granulomatous myocarditis and epicarditis were associated with systemic dissemination or spread from adjacent lung or air sac. Granulomatous nephritis, tubulonephritis, and nephrosis were frequently associated with intralesional gout tophi, seen as a radiating, amphophilic, crystalline material within tubular lumens and/or granulomas (Figure 1c).

Hemorrhagic enteritis followed by granulomatous enteritis were the most frequent findings in the alimentary system (29.4 and 17.6%, respectively). Lesions were multifocal and segmental. Minimal periganglionar inflammation associated with proventricular thinning of the wall was observed in two *Psittacus erithacus* (11.7%).

The liver was the most affected organ. Multifocal necrotizing hepatitis with or without associated hemorrhage and inflammation was observed in 23.5% of the cases. Hepatic granulomas were multifocal and similar to those described in the lung. Acid-fast bacteria consistent with mycobacteria were not detected in any of the cases. Bile stasis within hepatocellular canaliculi in the absence of obstruction was observed in 29.4% of the birds and considered an indicator of hypo/anorexia.

In reptiles, pulmonary interstitial edema and granulomatous pneumonia were the most relevant findings (Figure 1d,e). Granulomas were mainly heterophilic, and mycobacteria (acid-fast bacilli) were not detected in any of the cases. In the cardiovascular system, lesions were mainly observed within the heart (granulomatous epi/myocarditis) and were usually associated with granulomas in other locations such as lung and liver, indicating sepsis.

In the kidneys, the majority of renal lesions (inflammatory, chronic fibrosis and degenerative) were associated with the presence of urate tophi (Figure 1f).

Bile stasis within hepatocellular canaliculi and hepatocellular atrophy were observed in over 50% of the cases and used as indicators of hypo/anorexia and possible cachexia.

### 3.2. Infectious Disease

#### 3.2.1. Bacterial and Fungal Infections

**Figure 2:**
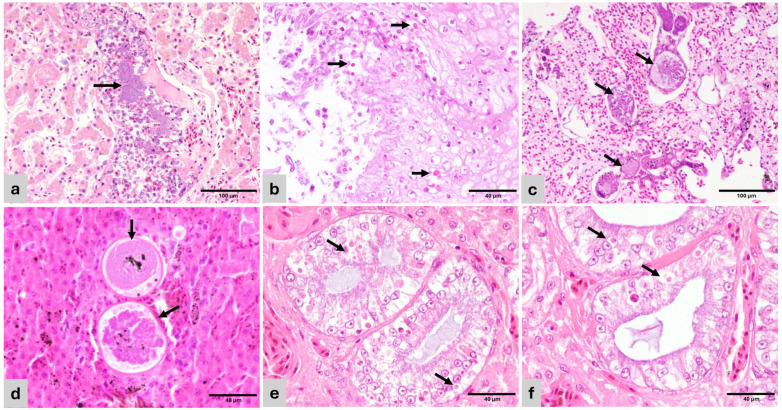
Histopathology of representative lesions (H&E). (**a**) *Varanus pilbarensis*. Liver—Dilated vein containing embolic bacterial colonies (arrow). Hepatocytes are atrophied and contain small amounts of cytoplasmic pigment (bile or hemosiderin). (**b**) *Amazona ochrocephala*. Esophagus—mucosal hyperplasia with hydropic degeneration and numerous superficial and intercellular yeasts consistent with *Candida* sp. (arrow). (**c**) *Spinus cucullatus*. Lung—The air capillary septae are expanded by 100–250 µm diameter thin-walled megaloschizont cysts containing merozoites (haemosporozoa) (arrows). Adjacent air capillary septae are edematous. (**d**) *Spinus cucullatus*. Liver—Haemosporozoa megaloschizonts in parenchyma (arrows). (**e**) *Ctenosaura* sp. Kidney—Collecting ducts with hypertrophied and degenerate epithelial lining, containing multiple eosinophilic, round, 4–10 µm inclusion bodies (arrows). (**f**) *Ctenosaura* sp. Kidney—Detail of tubular epithelium with inclusion bodies (arrows).

In birds, bacteria and bacterial sepsis were the cause of the majority of pulmonary and hepatic granulomas. The multifocal distribution of the granulomas obliterating or surrounding blood vessels suggested a hematogenous spread of the inciting cause. Similar findings were observed in reptiles. Bacterial colonies were more frequent within granulomas in reptiles than in birds (Figure 2a), and Gram negative in most cases. Acid-fast stains were negative in all cases, discarding mycobacteria.

The hemorrhagic enteritis in birds was associated with either intralesional bacterial colonies or hematogenous granulomatous spread. Therefore, they are presumed to be bacterial in origin.

A *Brachylophus vitiensis* with a history of colonic prolapse had a segmental, transmural, ulcerative colitis with numerous small colonies of cocci. The inflammation had extended into the adjacent coelomic adipose tissue, causing coelomitis and sepsis.

*Candida* sp. was associated with ulcerative and proliferative esophagitis in the *Amazona ochrocephala* (Figure 2b). This bird also had concomitant hemorrhagic enteritis.

#### 3.2.2. Parasitic Disease

The *Spinus cucullatus* had granulomatous pneumonia and hepatitis with intralesional, large, 100–250 µm diameter thin-walled cysts containing myriad merozoites, resembling megaloschizonts of unidentified haemosporozoa, similar to *Leucocytozoon* sp. (Figure 2b,c).

The *Lonchura oryzivora* had a granulomatous enteritis with intralesional flagellated protozoa within intestinal crypts. Sections of protozoa were up to 12 µm long and were pyriform, binucleate, and uniflagellate. Morphology was consistent with *Giardia* sp. or *Hexamita* sp.

Protozoa within intestinal crypts were also observed in an *Ara chloropterus* with granulomatous enteritis. In this case, organisms were round, 7–10 µm in diameter, and overlay the apical surface of enterocytes, making them compatible with *Cryptosporidium* sp.

#### 3.2.3. Viral Disease

The mild lymphoplasmacytic periganglioneuritis within the proventricular wall in two of the *Psittacus erithacus*, together with dilation and overall cachexia, was suspicious of proventricular dilation disease (PDD), a syndrome associated with bornavirus infection in Psittacine birds.

Embolic necrotizing hepatitis in the absence of intralesional infectious agents was suspicious of viral disease in an *Ara chloropterus* and a *Psittacus erithacus*.

Unidentified intracytoplasmic, round, 4–10 µm, eosinophilic inclusion bodies were observed in the renal tubular epithelium of a *Ctenosaura* sp. Inclusion bodies were associated with necrosis, apoptosis, and adjacent inflammation and were not observed in any other epithelia. Inclusions were PAS-negative discarding saccharides or other storage components (Figure 2e,f).

### 3.3. Non-Infectious Disease

Tubulonephrosis and granulomatous nephritis associated with urate tophi were observed in three birds (*Amazona aestiva*, *Ara chloropterus,* and a *Momotus momota*). Visceral gout, also affecting kidneys, was seen in 6 reptile specimens. Other sites with tophi and associated granulomatous and granulocytic infiltrates were mainly the heart and coelomic adipose tissue. In both birds and reptile specimens with visceral and/or renal gout, there was additional evidence of anorexia or dehydration (hepatocellular atrophy, bile stasis, muscle atrophy).

Secondary AA amyloidosis was observed in the liver of a *Psittacus erithacus* and in two reptiles (*Ctenosaura similis* and *Brachylophus vitiensis*).

### 3.4. Cause of Death

The cause of death was determined in 14 of the 17 birds (Table 4). In 10 birds, mortality was a consequence of infectious disease. Bacterial infection with septic complications accounted for 17.6% of bird deaths. In sepsis, multiple organs were affected, mainly lungs and liver, as well as kidneys and intestine to a lesser extent. Protozoal infection causing severe systemic disease (in the case of *Leucocytozoon* sp.) and granulomatous enteritis (associated with enteric flagellated protozoa or *Cryptosporidium* sp.) accounted for another 17.6% of deaths.

Lesions directly or indirectly associated with a viral infection were death-related in 23.3% of birds.

A probable cause of death was identified in 11 of the 12 reptiles. Bacterial sepsis accounted for the majority of deaths (41.5%), and in several cases, it coexisted with renal or visceral gout. An infectious nephritis of unknown origin (viral or protozoal) was associated with mortality in a *Ctenosaura* sp.

## 4. Discussion

There have been many surveys in recent years providing valuable information on diseases and mortality of birds and reptiles maintained in captivity in zoological collections or rehabilitation centers throughout the world [8,10,11,12]. However, taxa-specific information regarding pathologies or medical management are often scarce or too general in scope. This lack of information adds to the challenge that many CITES-designated rescue centers must face when taking in live specimens of variable taxa to rehabilitate and maintain for unknown and often prolonged periods of time [4].

The information obtained in the present postmortem survey provided insights on diseases and their origin, pathogeneses, and environmental/management hazards in a confiscated population of birds and reptiles maintained in a controlled environment at a FIEB research center.

This study revealed that bacterial infections and subsequent septicaemia was a major systemic complication in both birds and reptiles and was the cause of death in the majority of the reptiles. Bacterial dissemination was determined by the presence of bacterial emboli and multifocal granulomas, with frequent intralesional bacterial colonies. Granulomas were consistently found in the liver, followed by the lung and coelomic cavity, the latter predominantly in reptiles. Comparable results have been reported in a larger survey analyzing birds from diverse backgrounds, both free-ranging and captive [10]. Septicaemia in reptiles is often associated with primary skin lesions [12]. Skin lesions in the present survey were limited to a *Uroplatus lineatus* with granulomatous dermatitis and septic complications. Gram positive coccoid bacteria were detected within cutaneous and systemic granulomas, presuming an association between the lesions and systemic haematogenous spread. Incidentally, a mild, non-specific granulomatous dermatitis was encountered in a *Cacatua alba* without major repercussion.

Acute hemorrhagic enteritis was additionally associated with secondary septic complications. The cause of the enteritis was not identified in any of the birds. However, similar findings are described in domestic birds such as chickens and turkeys and can be caused by or associated with primary *Clostridium* sp. or *Escherichia coli* infections [13], or viral diseases caused by coronavirus or adenoviruses [13,14]. Viral inclusions were not observed in the gastrointestinal tract of any of the cases. However, secondary bacterial infections affecting the gastrointestinal tract have previously been reported by Nemeth *et al.* [10], reporting secondary bacterial infections in the gastrointestinal tract. One of the birds was a *Psittacus erithacus* with suspicion of proventricular dilation disease, an entity associated with bornaviral infection and a known cause of emaciation and immunocompromise [15,16], a situation that would favor a secondary bacterial co-infection as the cause for the enteritis.

Viral diseases were suspected in a small number of animals, particularly in the birds, however complementary serology or PCR analysis were either unavailable or inconclusive. Subclinical PDD was suspected in two *Psittacus erithacus*. Both psittacines had a history of poor body condition and damaged plumage, anorexia, or difficult ingestion. Minimal periganglionar lymphoplasmacytic infiltrates were observed in a moderately thinned proventricular wall. Although postmortem commercial PCR trials were negative for bornavirus, results were not considered conclusive given that trials were performed using organ tissues and not the recommended cloacal swabs or bird droppings [16]. One of the birds died of severe hemorrhagic enteritis (as mentioned earlier) while the other, in addition to emaciation had severe necrotizing hepatitis. An undetermined opportunistic viral infection was suspected, secondary to immunosuppression.

Granulomatous enteritis was associated with protozoa in several birds. Structures morphologically consistent with flagellated protozoa (*Giardia* sp.- or *Hexamita* sp.-like organisms) or *Cryptosporidium* sp. were associated with these lesions. These infections cause severe economic loss in poultry, but their role in wild and/or exotic birds is incompletely understood [17]. Surveys suggest that wild and exotic species may act as reservoirs involved in transmission and spread, with subclinical or no clinical disease [18]. In both cases in our study, intense inflammation of the affected intestinal tract together with dehydration and cachexia suggested clinical disease was associated with the parasitic infection. A *Spinus cucullatus* had a severe pneumonia associated with unidentified haemosporozoan megaloschizonts and hepatic dissemination. Microgamonts or macrogametes were not observed in peripheral blood cells. Birds are definitive hosts for many species of haemosporozoa and other vector-borne parasites, and they may also act as reservoirs [19]. Little could be elucidated about the life cycle of this parasite and its relevance, aside from the fact that it was associated with disease and mortality in this case.

It is interesting to highlight the discovery of intracytoplasmic eosinophilic inclusion bodies in the lumen and epithelium of renal collecting ducts and tubules of a female *Ctenosaura* sp. Inclusions were PAS and Giemsa negative and a viral entity was pondered as one of the plausible causes. The location and morphology of the inclusions bared some resemblance to coronavirus or nidovirus but also shared features with paramyxoviral inclusions or with inclusion body disease in boids, a disease caused by a reptarenavirus [20,21,22,23]. Reptarenaviruses and paramyxoviruses have been commonly isolated and reported in Ophidia [21,22]. A novel nidovirus associated with necrotizing nephritis was isolated in a mass mortality event of Bellinger River snapping turtles in Australia [20]. Other differentials considered were protozoa. *Entamoeba invadens*, *Cryptosporidium* sp., and *Eimeria* sp. have been reported in the urinary tract of certain reptiles, particularly chelonians and snakes [24]. Further analyses including electron microscopy and PCR are warranted in order to identify these organisms and understand the role played in the renal disease and death of the animal. These findings were yet another example of the relevance of these surveys that provide new findings and insights on infectious entities with uncertain impact on health and transmission.

Non-infectious diseases were also a part of this survey, with a special mention to renal and visceral gout, particularly amongst reptiles. Visceral gout was the cause of death in 25% of reptiles. Animals with visceral gout also had severe renal gout. Secondary gout associated with dehydration is common in reptiles [24,25] and was presumed to be the cause of gout in this survey. Dehydration results in hyperuricemia. Uric acid is cleared through the renal tubules and, in excess, crystalizes and deposits within tubules, causing necrosis and secondary inflammation [25]. Other causes of renal or systemic gout such as high-protein diets or drug administration were ruled out with the clinical history.

Amyloidosis was observed in two reptiles and a bird, although it was considered the cause of death in only one of the reptiles and the bird. Pathologic deposition of serum-associated amyloid, known as secondary AA-amyloidosis, is the form of amyloidosis reported in reptiles and birds and is commonly associated with chronic inflammatory disease in animals [26]. A recent study in snakes suggested that amyloidosis may be an aging change rather than being associated with inflammation or disease in reptiles [27]. In the present survey, amyloid deposits were observed in the liver of a *Ctenosaura* sp. and a *Brachylophus vitiensis*. The *Ctenosaura* sp. had no evidence of associated chronic disease or inflammation, which could support this new hypothesis. However, the *Brachylophus vitiensis* had a history of recurrent colonic prolapse and associated chronic inflammation. Amyloidosis was detected in a single *Psittacus erithacus* with chronic enteritis.

Pulmonary proteinosis was observed in a *Lorius chlorocercus*. This rare entity consists of eosinophilic crystalline material within tertiary bronchi and air capillaries and in the present case was associated with interstitial fibrosis. The cause is unknown and considered incidental in birds [28], unlike in humans where both primary and secondary forms are described, the first being an autoimmune disorder and the latter being secondary to infection or toxin inhalation [29]. The significance and origin of the lesion were undetermined; however, given the large proportion of affected parenchyma and the associated fibrosis, certain degree of respiratory distress was considered plausible. Findings showed certain similarities with the human secondary counterpart. An unrelated trauma was suspected as the underlying cause of death.

Results showed that in general disease transmission was limited in both reptiles and birds. None of the deaths were coincident in time aside from the *Psittaccus erithacus* with suspicion of PDD. Transmission of avian bornavirus (ABV) is incompletely understood, though it is known to be contagious [30]. Spread of disease has been documented in aviaries after the introduction of affected birds and a fecal–oral route of transmission is considered the most plausible route [30,31]. Screening of ABV was performed routinely in *Psittaccus erithacus* during quarantine and before introducing the animals to their conspecifics in a definitive enclosure. Both animals tested negative yet showed clinical and postmortem signs of chronic disease. Given viral shed is intermittent it was speculated that contact with an infected animal was plausible and went undetected. Screening protocols have henceforth been reinforced in the permanent population as well.

Bacterial infections were independent, scattered in time and amongst separate enclosures and species, which disregarded horizontal transmission. Bacterial infections are commonly traced to poor hygiene, overcrowding, or immunosuppression, and special emphasis is placed on care and management of enclosures [5,7,32]. Stressful situations or environments are factors that can compromise overall immunity. Unnecessary handling, excessive movement or introduction of new individuals may trigger stress and predispose disease [5,32].

Anorexia and dehydration can predispose gout and secondary renal disease in reptiles [25]. This was the primary cause behind visceral and renal gout in reptiles within this study. Careful monitoring of animal movements and eating habits could help early detection of disease, debilitation, or situations predisposing dehydration.

Rescue centers such as FIEB struggle to provide adequate housing, management, and care to confiscated animals for extended periods of time as well as flexible adaptations of environments and enclosures. These facilities must prepare for the reception of diverse taxa, often with unknown requirements or very high sensitivity to captivity, leading to extraordinary efforts to safeguard the welfare of the animals.

Our results suggest that a more individualized health plan targeting specific individuals or small groups could improve early detection of disease and treatment, rather than targeting populations, which is the more commonly applied strategy in other types of zoological facilities. However, an individualized approach is not always feasible given the particularities of rescue centers that are often understaffed or lack sufficient economic resources to face unpredictable challenges. Along this line, molecular/microbiological screening of infectious agents, blood tests, and fecal screening for parasites are beneficial for prevention and early detection when available.

This study highlights the complexity of disease processes affecting confiscated birds and reptiles in CITES rescue settings and provides invaluable information for other rescue centers that may impact the success of conservation strategies. Bacterial septicaemia emerged as an important cause of mortality, alongside a diversity of infectious and non-infectious conditions. The findings underscore the multifactorial nature of disease in captive wildlife, where stress, dehydration, immunosuppression, and limited species-specific knowledge play a critical role in health outcomes. Importantly, this study demonstrates the value of systematic postmortem investigations in identifying emerging or poorly understood pathologies and informing management practices. Enhanced surveillance, individualized health monitoring when feasible, and strengthened biosecurity and husbandry protocols are essential to improving welfare and survival in CITES-designated rescue and rehabilitation centers.

## Figures and Tables

**Table 1:** Confiscated bird and reptile species between 2021 and 2024 and the number of individuals, sex, and CITES Appendix listing (https://cites.org/eng/app/appendices.php; access date: 18 March 2026).

**Birds**			
**Species**	***n***	**Male/Female/Unknown**	**CITES Appendix**
*Psittacus erithacus*	5	2/1/2	Appendix I
*Cacatua alba*	2	1/1/0	Appendix II
*Agapornis fischeri*	1	0/1/0	Appendix II
*Amazona aestiva*	1	1/0/0	Appendix II
*Amazona ochrocephala*	1	1/0/0	Appendix II
*Ara chloropterus*	1	1/0/0	Appendix II
*Lonchura oryzivora*	1	0/1/0	Appendix II
*Lorius chlorocercus*	1	0/1/0	Appendix II
*Momotus momota*	1	0/0/1	Not included *
*Psittacula cyanocephala*	1	1/0/0	Appendix II
*Spinus cucullatus*	1	1/0/0	Appendix I
*Strix rufipes*	1	0/1/0	Appendix II
**Reptiles**			
**Species**	***n***	**Male/Female/Unknown**	**CITES Appendix**
*Brachylophus vitiensis*	2	1/0/1	Appendix I
*Uroplatus lineatus*	2	1/1/0	Appendix I
*Varanus pilbarensis*	2	1/1/0	Appendix II
*Brachylophus fasciatus*	1	1/0/0	Appendix I
*Ctenosaura* sp.	1	0/1/0	Appendix II
*Ctenosaura similis*	1	0/0/1	Appendix II
*Stigmochelys pardalis*	1	0/1/0	Appendix II
*Uromastyx* sp.	1	0/0/1	Appendix II
*Varanus salvator*	1	0/1/0	Appendix II

* Not included in CITES Appendix I or II but subjected to strict regulations. Commission Regulation (EU) 2017/160.

**Table 2:** Birds: List of organ systems examined histologically and relevant lesions in each system, n, and percent of affected birds.

System	Lesion	N	%
Integument	Granulomatous dermatitis	1	5.8
	Hyperkeratosis	1	5.8
Respiratory	Tracheal hemorrhage	1	5.8
	Granulocytic tracheitis	1	5.8
	Pulmonary interstitial edema and congestion	8	47
	Granulomatous pneumosaculitis	1	5.8
	Granulomatous pneumonia	4	23.5
	Pulmonary proteinosis with fibrosis	1	5.8
Cardiovascular	Heterophilic granulomatous myocarditis	1	5.8
	Myocardial fibrosis	2	11.7
	Myocardial necrosis	1	5.8
	Heterophilic granulomatous epicarditis	1	5.8
	Myocardial hemorrhage	1	5.8
	Arterial and valvular cartilaginous metaplasia	1	5.8
Spleen/Lymphoid	Periellipsoidal hyperplasia	2	11.7
	Granulomatous splenitis	1	5.8
	Lymphoid hyperplasia	1	5.8
	Periarteriolar hyperplasia	1	5.8
	Hemosiderosis	1	5.8
Renal	Granulomatous tubulonephritis	2	11.7
	Membranous glomerulonephritis	2	11.7
	Heterophilic nephritis	3	17.6
	Interstitial granulomatous nephritis	3	17.6
	Tubulonephrosis	1	5.8
	Glomerulosclerosis	1	5.8
Alimentary	Granulomatous enteritis	3	17.6
	Lymphoplasmacytic enteritis	1	5.8
	Hemorrhagic enteritis	5	29.4
	Lymphoplasmacytic periganglioneuritis	2	11.7
	Ulcerative esophagitis	1	5.8
	Granulomatous ventriculitis	1	5.8
	Hemorrhagic proventriculitis	1	5.8
Liver	Necrotizing hepatitis	4	23.5
	Hepatic hemorrhage	2	11.7
	Amyloidosis	1	5.8
	Granulomatous hepatitis	2	11.7
	Hepatocellular degeneration	2	11.7
	Bile stasis	4	23.5
	Biliary hyperplasia	1	5.8
	Hepatocellular atrophy	1	5.8
	Heterophilic hepatitis	5	29.4
Central Nervous System	Cerebral congestion	1	5.8

**Table 3:** Reptiles: List of organ systems examined histologically and relevant lesions in each system, n, and percent of affected reptiles.

System	Lesion	N	%
Integument	Granulomatous dermatitis	1	8.3
Respiratory	Interstitial edema and congestion	2	16.6
	Foveal hemorrhage	1	8.3
	Granulomatous pneumonia	1	8.3
	Foveal septae interstitial fibrosis	1	8.3
Cardiovascular	Granulomatous myocarditis	2	16.6
	Myocardial necrosis	1	8.3
	Granulomatous endocarditis	1	8.3
	Granulomatous epicarditis	1	8.3
Spleen/Lymphoid	Reticuloendothelial hyperplasia	1	8.3
	Granulomatous splenitis	1	8.3
	Lymphoid hyperplasia	1	8.3
Renal	Granulomatous nephritis	1	8.3
	Chronic interstitial nephritis	1	8.3
	Tubulonephrosis	5	41.6
Alimentary	Granulomatous enteritis	1	8.3
	Proliferative gastritis	1	8.3
Liver	Perivascular amyloidosis	2	16.6
	Granulomatous hepatitis	1	8.3
	Hepatic fibrosis	1	8.3
	Bile stasis	6	50
	Hepatocellular atrophy	8	66.6

**Table 4:** Causes of mortality, n, and percent of birds and reptiles that died from each cause.

Cause of Death	Birds (n)	%	Reptiles (n)	%
Bacterial disease and sepsis	3	17.6	5	41.5
Gout	1	5.8	4	33.3
Amyloidosis	1	5.8	1	8.3
Protozoal infection	3	17.6	0	0
Viral infection	4	23.3	0	0
Infectious nephritis	0	0	1	8.3
Cachexia	1	5.8	0	0
Trauma	1	5.8	0	0
Undetermined	3	17.6	1	8.3

## Data Availability

The original contributions presented in this study are included in the article. Further inquiries can be directed to the corresponding author.

## Abbreviations

The following abbreviations are used in this manuscript:

ABV	Avian bornavirus
CITES	Convention on International Trade in Endangered Species of Wild Fauna and Flora
EAZA	European Association of Zoos and Aquaria
FIEB	Foundation for Research in Ethology and Biodiversity
HVCV	Complutense Veterinary Teaching Hospital
H&E	Hematoxylin and Eosin
PAS	Periodic Acid-Shiff
PCR	Polymerase chain reaction
PDD	Proventricular dilation disease
ZN	Ziehl-Neelsen
